# Supplementary material for: HIVprotI: an integrated web based platform for prediction and design of HIV proteins inhibitors
Source: J Cheminform. 2018 Mar 9;10:12. doi: 10.1186/s13321-018-0266-y (PMC5845081; doi:10.1186/s13321-018-0266-y)
Supplement: Supplementary file 2 — Additional file 2. Source code of HIVProtI web server. [file 13321_2018_266_MOESM2_ESM.zip › HIVprotI_Source-code/hivprotI/weka-3-6-6/doc/weka/classifiers/bayes/net/search/local/package-frame.html]

weka.classifiers.bayes.net.search.local


weka.classifiers.bayes.net.search.local

Interfaces 
  
*Scoreable*

Classes 
  
GeneticSearch
  
HillClimber
  
K2
  
LAGDHillClimber
  
LocalScoreSearchAlgorithm
  
RepeatedHillClimber
  
SimulatedAnnealing
  
TabuSearch
  
TAN
